# Influence of neuromarketing on the increase in shopping anxiety in women in the city of santa marta

**DOI:** 10.1192/j.eurpsy.2024.1672

**Published:** 2024-08-27

**Authors:** K. L. Perez Correa, A. Guardiola Esmeral

**Affiliations:** ^1^Magdalena, Universidad Cooperativa de Colombia, Santa Marta, Colombia

## Abstract

**Introduction:**

New technological trends and access to more information have generated an anxious disorder and the need to obtain everything that the consumer society has to offer, this has increased with the influence of neuromarketing in internet ads.

**Objectives:**

The objective that was raised in the present investigation was to analyze the influence of neuromarketing in the increase of anxiety reflected in compulsive purchases of women in the city of Santa Marta.

**Methods:**

The field research design is non-experimental and cross-sectional, the sample taken was of 500 women with purchasing power of more than three Colombian minimum wages.

The IDARE Ch. Spielberger, R. Díaz Guerrero et al. (1966) checklist was applied; To review the relationship between advertising with neuromarketing, anxiety and compulsive purchases, a Likert-type scale instrument was designed and validated with the Alpha Cronbach Coefficient. Analysis of Covariance ANOVA, inferential statistics and SPSS were performed.

**Results:**

57% of the women meet the criteria for the IDARE clinic. The analysis of the questionnaire showed a goodness of fit of R² = 0.697. The result indicates that the more hours women spend on the internet with access to ads focused on neuromarketing, the more they feel the need to buy, and this generates anxiety processes.

**Conclusions:**

The mental triggers used by neuromarketing accelerate the need in women to buy the solutions that they sell and the same need to buy is evident in the signs of anxiety that is reflected in the women under study. Training that educates women to spend less time connected to the Internet is recommended, but it is also essential that they understand that advertising and marketing exert pressure that increases their anxiety and need to purchase, so it is recommended the implementation of training in personal management and control.

**Disclosure of Interest:**

None Declared

